# Microbial Dysbiosis in the Skin Microbiome and Its Psychological Consequences

**DOI:** 10.3390/microorganisms12091908

**Published:** 2024-09-19

**Authors:** Alejandro Borrego-Ruiz, Juan J. Borrego

**Affiliations:** 1 Departamento de Psicología Social y de las Organizaciones, Universidad Nacional de Educación a Distancia (UNED), 28040 Madrid, Spain; a.borrego@psi.uned.es; 2 Departamento de Microbiología, Universidad de Málaga, 29071 Málaga, Spain

**Keywords:** skin microbiome, skin diseases, mental disorders, psychological consequences

## Abstract

The homeostasis of the skin microbiome can be disrupted by both extrinsic and intrinsic factors, leading to a state of dysbiosis. This imbalance has been observed at the onset of persistent skin diseases that are closely linked to mental health conditions like anxiety and depression. This narrative review explores recent findings on the relationship between the skin microbiome and the pathophysiology of specific skin disorders, including acne vulgaris, atopic dermatitis, psoriasis, and wound infections. Additionally, it examines the psychological impact of these skin disorders, emphasizing their effect on patients’ quality of life and their association with significant psychological consequences, such as anxiety, depression, stress, and suicidal ideation in the most severe cases.

## 1. Introduction

The skin is a dynamic tissue composed of multiple cells and structures that work together to maintain the integrity of the body against external challenges. The main skin layers are the epidermis and the dermis, which have several roles, such as defense barrier, homeostatic functions, wound repair, support of vitamin D synthesis, and avoidance of colonization by pathogenic organisms [1,2]. Human skin constitutes a true microbial ecosystem, termed the skin microbiome (SM), consisting of bacteria, fungi, and viruses. However, skin varies in its physicochemical and biochemically properties along body sites, giving rise to distinct microenvironments within the SM that promote specific microbial populations [3]. Thus, sebaceous sites of skin have a high density of lipid-rich sebaceous glands, which promote colonization by lipophilic microbial taxa, such as the bacteria *Cutibacterium* (formerly *Propionibacterium*) and the fungi *Malassezia* [1,4]. Moist areas have elevated concentrations of apocrine sweat glands and are primarily populated by the bacterial genera *Corynebacterium* and *Staphylococcus* [4]. Conversely, dry skin sites harbor the lowest levels of microbial abundance and diversity, with *Corynebacterium*, *Cutibacterium*, and *Streptococcus* being the dominant bacterial species [5]. However, disruption of the cutaneous barrier, as happens with skin aging, disease, and injuries, can lead to microbial dysbiosis and heighten the risk of skin infections, such as acne vulgaris, atopic dermatitis (AD), psoriasis, and wound infections [6].

The psychological consequences of skin disorders often include elevated levels of anxiety and depression. In this respect, there is a bidirectional relationship between dermatological conditions and mental health, with skin disorders exacerbating psychological distress and vice versa [7].

Therefore, in the present narrative study, we review recent findings on the relationship between the SM and the pathophysiology of various skin diseases, including acne vulgaris, AD, psoriasis, and wound infections. Additionally, we examine the psychological consequences associated with these skin conditions.

## 2. Human Skin Microbiome

The skin is the largest organ of the human body (average surface area of 30 m^2^) and harbors 10^3^ to 10^6^ microorganisms in an adult, depending on the sampling site [8]. Most of the bacteria of the human SM are included in four phyla: Actinomycetota, Bacillota, Pseudomonadota, and Bacteroidota, and the dominant genera are *Staphylococcus*, *Cutibacterium*, *Corynebacterium*, *Micrococcus*, *Streptococcus*, and *Acinetobacter* [9]. In addition, fungi belonging to the divisions Ascomycota and Basidiomycota also form part of the SM. The dominant genus is *Malassezia*, with the species *M. restricta*, *M. globosa*, and *M. sympodialis*, although the highest fungal diversity has been observed on the feet, colonized by the genera *Aspergillus*, *Cryptococcus*, *Epicoccum*, and *Rhodotorula* [10]. Viruses belonging to the families *Papillomaviridae*, *Polyomaviridae*, *Poxviridae*, and *Circoviridae* have been reported as common members of the SM [1,11]; although several bacteriophages have also been recently characterized [11,12].

An indicator of a healthy balance between skin microbial communities (eubiosis) is the preservation of skin barrier integrity, which is achieved by the skin cell development [13], the production of antimicrobial peptides by tissue-resident immune cells [14,15], and the prevention of pathogen overgrowth through microbial competitive interactions [16,17]. Perturbation of this balance (dysbiosis) is marked by the excessive growth of certain bacterial species and by a reduction in community diversity. Such dysbiosis can result in compromised wound healing, heightened inflammation, and an increased risk of microbial infections [3,18].

### Changes in the SM with Age

The homeostasis of the SM shifts with age because of lifelong changes in skin physiology as the cutaneous immune system develops and hormones stimulate the growth of sweat and sebum glands, affecting the availability of essential nutrients for microorganisms. At birth, the skin, like in the gut, is rapidly colonized from the immediate environment; therefore, the mode of delivery influences the initial composition of the community [19]. For instance, the SM of vaginally delivered neonates is predominantly comprised of vaginal-associated microbiota, mainly *Lactobacillus* and *Prevotella*, and shows a higher presence of the yeast *Candida albicans*. Newborns delivered by cesarean section possess microbiomes that contain microorganisms from maternal skin, such as *Corynebacterium*, *Cutibacterium*, *Staphylococcus*, and *Streptococcus* [20]. Newborn and infant skin begins to acidify and has a higher water content, elevated pH, reduced sebum production, accelerated epidermal turnover, and enhanced antimicrobial properties [21]. Decreased sebum production early in life is linked to a lower abundance of *Corynebacterium*, *Cutibacterium* and *Malassezia*, to an increase in staphylococci and streptococci, and to a mycobiome predominantly featuring *Candida* species [5,20,22].

Commensal colonization also promotes the maturation of immune cells, especially the localization of regulatory T cells (Treg) to developing hair follicles shortly after birth [23]. The precise interaction between commensal bacterial antigen and Tregs is necessary to establish immune tolerance, which precludes inflammation directed at the commensals over time [23,24]. Concurrent recognition of several pathogen-related molecules and microbial toxins during initial colonization events restricts the formation of pathogen-specific Tregs [25]. 

Pubertal hormones (mainly androgens) lead to physical and sexual development that also directly foster functional and structural modifications in the skin, such as sebaceous glands, which increases sebum production, and apocrine sweat gland development, which increases body odor. These changes lead to subsequent shifts in microbial composition [26]. Children in the prepubertal stage exhibit higher relative abundances of Bacteroidota, Pseudomonadota, and *Streptococcus*, along with greater bacterial and fungal diversity compared to young adults [27,28]. As mentioned previously, the young adult SM is dominated by lipophilic microorganisms, including *Corynebacterium*, *Cutibacterium*, and *Malassezia* [27,28], a profile related to increased sebum production and to elevated serum hormone levels [28]. Estrogen and progesterone strengthen the skin barrier and support wound healing by promoting collagen synthesis and stimulating keratinocyte proliferation. Additionally, they regulate immune function in both pro- and anti-inflammatory manners [3,29]. These hormonal-driven alterations in skin physiology result in changes to the skin microbial communities [28]. Interestingly, androgen-driven development of sebaceous gland and estrogen-induced changes in skin surface lipids [30] fosters the predominance of lipophilic species, such as *Cutibacterium acnes* and *Malassezia restricta* [28]. While apocrine gland secretions are initially odorless, body odor in children and adolescents is linked to microbial production of isovaleric and acetic acids (with a sour smell), and of sulfur compounds (with a rotten-egg smell) [31]. In young adults, body odor, particularly from the axillary area [31], is related to the breakdown of sebum by *Corynebacterium* species into volatile fatty acids (with a cheesy smell) and sulfanyl alkanols (with an oniony smell) [32]. 

Sex differences in skin microbial composition become increasingly evident as puberty progresses [28]. During this period, the microbial communities in females tend to exhibit reduced diversity and a higher predominance of *Cutibacterium*, while the SM of males show greater inter-individual variability and increased diversity, characterized by elevated levels of *Corynebacterium*, *Haemophilus*, *Staphylococcus*, and *Streptococcus* [28]. Furthermore, hormonal changes affecting skin physiology (such as a reduced pH and increased thickness) and immune system modifications during puberty likely contribute to various of the shifts observed in the SM [27]. The adult SM is stable over time, establishing networks of interactions between different microorganisms [33]. Each body site has a unique microenvironment (with a characteristic pH and nutrient pool) and a particular sweat and sebaceous gland density, which determines the prevailing microbial composition [34]. However, the SM may be influenced by the environment, geographic localization, and residence in the same localization [35]. 

As the skin ages, it undergoes several physiological changes caused by intrinsic and extrinsic factors. Intrinsically aged skin, impacted by genetic, hormonal, and metabolic modifications (e.g., decrease in estradiol levels), is characterized by diminished sebaceous gland activity, reduced blood circulation, and the breakdown of collagenous and fibrous extracellular matrices, resulting in atrophy, decreased lipid content, xerosis, and loss of skin cell regeneration [36,37]. In contrast, extrinsic factors are triggered by environmental factors, mainly UV exposure, and are manifested by telangiectasia, hyperpigmentation, pronounced wrinkles, and a leathery texture [38]. Both intrinsically and extrinsically aged skin exhibits a higher pH, reduced hydration, and lower expression of tight junction proteins compared to youthful skin. 

These changes can manifest as impaired wound healing, and as an increase in the production of natural moisturizing factors (NMFs), which are produced as a consequence of the loss of the skin’s ability to retain water. NMFs can foster bacterial growth and adherence to the skin [39] and are also associated with an increased abundance of several microbial taxa, such as *Anaerococcus*, *Corynebacterium*, *Micrococcus*, and *Streptococcus* [34], and with a reduction in *Cutibacterium* [34,39]. The decrease in sebocyte area and sebum generation after menopause in women is related to a loss of *Cutibacterium* and to a higher abundance of *Acinetobacter, Corynebacterium,* and *Streptococcus* [32,36,40]. In contrast, men have a significantly slower decline in sebum secretion and maintain a higher *Cutibacterium* abundance [41].

Elderly subjects maintain a low-grade inflammatory state with elevated systemic levels of pro-inflammatory cytokines [42]. Skin Langerhans cells are progressively lost from the epidermis [43], and cutaneous dendritic cells, in turn, show a diminished capacity for migrating to lymph nodes and present antigens to T cells [44]. This failure in antigen presentation leads to slower immune responses, reduced antimicrobial activity, and compromised wound healing [45]. The weakened immune defenses and higher prevalence of potentially pathogenic bacteria contribute to a significantly greater risk of skin infections in the elderly and to difficulties in resolving these infections [45,46].

## 3. Skin Microbiome in Disease

**Figure 1:**
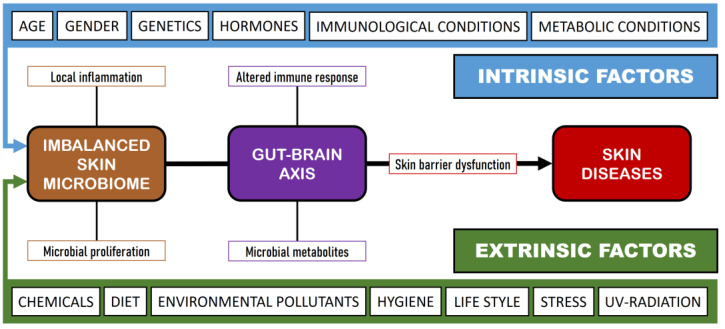
Influence of intrinsic and extrinsic factors on skin diseases.

A variety of intrinsic and extrinsic factors can influence the shape and composition of the SM, resulting in a state of dysbiosis [47] (Figure 1). While the mechanisms underlying dysbiosis in these diseases remain poorly understood, there is a continuing debate about whether dysbiosis acts as a cause or as a result of the disease [6]. In this situation, the commensal skin microbiota is unable to prevent colonization by pathogenic microorganisms, a phenomenon termed colonization resistance [48]. The mechanisms through which colonization resistance take place have become the focus of current research, involving not only competition for adhesion sites and nutrients but also the role of signaling molecules. A dysbiosis process has been noted at the onset of skin diseases, such as acne vulgaris, AD, psoriasis, and wound infections.

### 3.1. Acne Vulgaris

Increased levels of androgens at the start of puberty can trigger fluctuations in skin cell activity, inflammation, and the colonization of hair follicles by *C. acnes*, leading to the onset of acne. Acne is a persistent inflammatory condition of the pilosebaceous follicle that affects over 85% of adolescents and young adults. Its pathogenesis encompasses elevated sebum production, follicular hyperkeratinization, clogged hair follicles, painful papules, and inflammation [49]. *C. acnes* is a lipophilic bacterium that predominantly inhabits sebaceous skin areas. In this regard, each pore is typically governed by a single, nearly clonal lineage [50] and individuals possess a distinctive combination of *C. acnes* strains [5]. Although acne has been primarily related to *C. acnes* proliferation, some researchers have reported that the levels of *C. acnes* do not show significant differences between individuals with acne and those without [51]. Consequently, it seems that the reduction in diversity among the six phylogenetic groups (IA1, IA2, IB1, IC, II, and III), with a predominance of IA1, and to a lesser extent IA2, rather than the proliferation of *C. acnes*, contributes to the onset of acne [52]. These acne-associated phylotypes tend to release pro-inflammatory cytokines, show increased porphyrin production [53], and exhibit excessive lipase activity, which subsequently attracts neutrophils and promotes hyperkeratosis [54]. In addition, *C. acnes* can also develop biofilms that elevate its virulence and its resistance to antimicrobial treatment [55]. Interestingly, *Staphylococcus epidermidis* inhibits *C. acnes* proliferation by means of the release of succinic acid from glycerol fermentation [56], and also diminishes *C. acnes*-induced skin inflammation by generating lipoteichoic acid, which suppresses keratinocyte production of Toll-like receptor (TLR) 2, interleukin (IL)-6, and tumor necrosis factor (TNF)-α [57]. In contrast, *C. acnes* restrains the proliferation of *S. epidermidis* by maintaining the acidic environment of the pilosebaceous follicle, hydrolyzing sebum triglycerides and releasing propionic acid [58]. On the other hand, other commensal microorganisms such as *Malassezia* also participate in the pathogenesis of acne, as this genus possesses a lipase more active than that of *C. acnes*, and may attract neutrophils and stimulate the liberation of pro-inflammatory cytokines from monocytes and keratinocytes [59].

### 3.2. Atopic Dermatitis

AD (eczema) is an inflammatory skin disease, characterized by dry, inflamed, itchy skin patches, affecting between 15 and 20% of children and 2–10% of adults [60]. AD arises from a sophisticated interplay of genetic susceptibility, barrier dysfunction, both innate and adaptive immune responses, and SM dybiosis [61]. As a result, affected skin areas have increased permeability, decreased water retention, high pH, and disturbed lipid structure [62,63], allowing *Staphylococcus aureus* to access deeper layers of the skin [60]. 

Altered keratinocytes and microbial antigens provoke immune responses [64], which further exacerbate barrier defects via the downregulation of filaggrin [65], disruption of tight junctions, and diminishment in stratum corneum lipids, thereby increasing skin susceptibility to *S. aureus* colonization [65]. Thus, a continuous cycle of impaired barrier function, augmented microbial and irritant infiltration into deeper layers of the skin, and heightened inflammation, followed by itching and additional skin damage, develops. Further alterations in the microbiome associated with AD involve a decrease in *C. acnes*, *Corynebacterium*, *Dermacoccus*, *Micrococcus*, and coagulase-negative staphylococci (e.g., *S. epidermidis* and *S. hominis*), coupled with an increase in *Streptococcus* and various *Malassezia* species [62,64,66]. These microbial changes do not seem to be permanent, with a reduction in community diversity and increased *S. aureus* presence before and during AD flare-ups, followed by a gradual return to baseline levels after the flare resolves [67]. Whether *S. aureus* initiates AD or prospers as a result of the disease is yet to be clarified. However, toxins and proteases produced by *S. aureus* exacerbate damage to the skin barrier and stimulate both adaptive and innate immune responses [68].

On the other hand, *S. epidermidis* seems to be an antagonist of *S. aureus* [69]. *S. epidermidis* maintains SM balance by incorporating innate immune mechanisms that regulate effector T-cell activity, and by performing an antimicrobial role via the secretion of IL-1α by dendritic cells and keratinocytes, thus restricting the capacity of pathogens to cause infections [70]. In addition, colonization by *Malassezia* spp. intensifies with the severity of AD, and its pathophysiological impact may stem from the stimulation of pro-inflammatory cytokines and autoreactive cells. This process can lead to elevated expression of TLR2 and TLR4 via the release of immunogenic proteins [60,68].

### 3.3. Psoriasis

Psoriasis is a chronic inflammatory disease with worldwide prevalence and a multifactorial etiology, including genetic and non-genetic factors such as diet, drugs, infections, smoking, and stress [71]. These factors induce immune dysregulation and trigger interactions among innate immune cells, adaptive immune cells and keratinocytes, being mediated by cytokines (including IL-6, IL-17, IL-22, IL-23, interferon [IFN], and TNF-α), and leading to the development of erythematous and scaly plaques [72,73,74]. 

In addition, the breakdown of immune tolerance to cutaneous microorganisms has also been suggested to be involved in the pathogenesis of psoriasis [75], such as species of the genus *Staphylococcus* (*S. aureus*, *S. pettenkoferi*, and *S. sciuri*) [75,76]. These species colonize psoriatic lesions, secrete enterotoxins and toxic shock syndrome toxin-1, and induce an inflammatory Th17 response responsible for maintaining keratinocyte proliferation [76]. *Streptococcus pyogenes* infections of the upper respiratory tract are also considered an important etiological factor in psoriasis. These bacterial infections induce T-cell activation and produce IFN-γ, which is elevated in patients with chronic plaque psoriasis lesions [77]. Besides, bacterial superantigens located in the dermal layers bind directly to human leukocyte antigen (HLA)-DR receptors on dendritic cells, macrophages, and keratinocytes, perpetuating inflammation [75]. 

Diverse studies suggest that the gut is a possible origin of the dysbiosis detected in psoriasis. The most commonly reported findings are a reduction in the gut microbiome of *Bacteroides* and *Akkermansia* spp. and an increase in members of the phyla Bacillota and Actinomycetota [72,78]. This intestinal dysbiosis leads to bacterial translocation and induction of inflammation, resulting in the perpetuation of the inflammatory response as seen in inflammatory bowel and Crohn’s diseases [75,79].

### 3.4. Chronic Wounds

Chronic wounds are typically categorized as burns, diabetic ulcers, malignant ulcers, pressure ulcers, venous ulcers, and pyoderma gangrenosum [80]. The wound healing process involves an interaction among endothelial cells, fibroblasts, keratinocytes, and Langerhans cells, and it is linked to processes such as hemostasis, inflammation, proliferation, and remodeling [81]. Wound healing is regulated by several cytokines and growth factors, including platelet-derived growth factor, transforming growth factors (TGF-β, TGF-α), fibroblast growth factor, and vascular endothelial growth factor, which stimulate angiogenesis, granulation tissue formation, and tissue repair [82]. Multiple bacterial colonization of wounds forms biofilms that delay wound healing by activating neutrophils and pro-inflammatory macrophages, leading to the accumulation of pro-inflammatory cytokines, cytolytic enzymes, and free oxygen radicals [83,84]. The unregulated immune environment of chronic wounds promotes bacterial proliferation, resulting in a vicious cycle of biofilm growth and ongoing inflammation [85]. The major bacterial genera involved in this process are *Acinetobacter*, *Enterobacter*, *Serratia*, and *Pseudomonas* (Gram-negatives), as well as *Staphylococcus*, *Streptococcus*, *Enterococcus*, and *Finegoldia* (formerly *Peptostreptococcus*) (Gram-positives) [86,87].

Chronic wounds are more common in older adults, as well as in patients with chronic mechanical stress, diabetes, malnutrition, obesity, vascular disease, or in those ones with a combination of these aspects [88]. The majority of chronic wounds are colonized by polymicrobial communities, including *S. aureus, Pseudomonas aeruginosa*, *Proteus mirabilis*, *Escherichia coli*, *Acinetobacter baumannii* and *Klebsiella pneumoniae* [84]. Anaerobic bacteria in chronic wounds include *Prevotella*, *Peptoniphilus*, *Finegoldia*, and *Anaerococcus* [89]. In diabetic ulcers *S. aureus* is the most common infecting species, although other causative organisms include coagulase-negative staphylococci, *Streptococcus*, *Enterococcus*, *Corynebacterium*, members of the *Enterobacteriaceae* family, *P. aeruginosa*, *Prevotella*, *Poryphyromonas*, and *Bacteroides fragilis* [86,90]. Some pathogens have been identified in the acute phase after burn injury, including *S. aureus*, *S. pyogenes*, *E. coli*, *P. aeruginosa*, and coagulase-negative staphylococci [91]. Venous leg ulcers are a common chronic wound condition susceptible to infection by *P. aeruginosa*, *P. mirabilis*, *Enterococcus*, *S. aureus*, *A. baumannii*, *Klebsiella*, and *E. coli* [84].

## 4. Association of Skin Diseases with Mental Disorders

The skin plays a central role in the psyche of people affected by external dermatologic disorders, such as acne, dermatitis, or wound ulcers, which can lead to psychological adverse states such as depression, low self-esteem, shame, and social appearance anxiety, thus affecting their quality of life. Quality of life is defined by the World Health Organization as “an individual’s perception of his or her position in life within the context of the culture and value systems in which he or she lives, and in relation to his or her goals, expectations, standards and concerns”, and it includes various aspects such as health status, wealth, freedom, education, safety, and social belonging [92]. Self-esteem is a subjective concept that assesses a person’s self-worth, confidence in their abilities, and qualities. Healthy self-esteem is central for a balanced life, characterized by self-understanding and healthy relationships with oneself and others. The concept of self-esteem is integral to Maslow’s Hierarchy of Needs, which states that self-esteem is essential to human motivation and growth toward self-actualization. Factors such as age, disability, genetics, illness, physical ability, cognition, and socioeconomic status influence self-esteem [93]. Social appearance anxiety is the fear of being judged negatively by others because of one’s appearance. It is associated with social anxiety disorder, which negatively affects the quality of life and is inversely related to self-esteem. Social appearance anxiety involves inconsistent beliefs about one’s appearance, leading to negative body image, personality issues, depression, and affective problems [94]. Therefore, it is plausible to affirm that skin diseases can influence the development of several mental disorders in patients. In the following sections, we have reviewed the major psychological disorders associated with acne, AD, psoriasis, and wound infections.

### 4.1. Acne Vulgaris

Acne is a risk factor for mental disorders, including anxiety and mood disorders, negative body image, poor individual self-esteem, interpersonal relationships, and even suicide attempts in all age groups [95,96]. In accordance with the literature, acne and stress have a bidirectional relationship. For example, stress can exacerbate the severity of acne, while problems resulting from acne, such as social isolation and lack of self-confidence, can lead to depression [97]. In this respect, diverse studies have pointed out that an increase in stress levels correlates positively with an aggravation of acne severity in a significant proportion of patients with acne [98,99]. However, a different study stated an absence of a direct correlation between self-reported stress levels and the severity of acne, which may highlight variations in how individuals manage stress [100]. The characteristics and main findings of recent studies (2018–2024) on the relationship between acne vulgaris and psychological disorders are depicted in Table 1.

### 4.2. Atopic Dermatitis 

AD is characterized by pruritus and by reappearing eczematous patches and plaques on the skin. It interferes with sleep and its visible nature can be responsible for social stigma, low self-esteem, social withdrawal, reduced quality of life, and psychological distress [118]. AD is also associated with an increased risk of alexithymia, anxiety, depression, obsessive compulsive disorder (OCD), and suicidal ideation [119,120]. Furthermore, Cao et al. [121] investigated the causal relationships between AD and psychiatric disorders by means of a bidirectional two-sample Mendelian randomization analysis and concluded that AD was related to a higher risk of attention deficit/hyperactivity disorder (ADHD), autism spectrum disorder (ASD), anorexia nervosa (AN), and bipolar disorder (BD). Table 2 describes the relationship between AD and mental disorders.

### 4.3. Psoriasis 

The prevalence of depression is typically greater in individuals with more severe psoriasis compared to those with milder forms of the disease, and is higher in women than in men [135]. Depression in psoriasis significantly reduces quality of life, including aspects such as sexual dysfunction, sleep disturbances, and addictions. The visible lesions associated with the physical burden of this disease can provoke adverse reactions and stigma, resulting in anxiety, depression, diminished self-esteem, and reduced subjective well-being [136].

On the question of whether psoriasis is the underlying cause of psychiatric comorbidities or if, conversely, these psychiatric conditions determine the progression of the skin disorder, Carrasco and Ballesca [137] proposed several explanations: (i) The connection between psoriasis and psychiatric disorders may involve genetic factors that could predispose individuals to both the skin condition and psychological or psychiatric states. Several investigations have identified common alterations in loci and single nucleotide polymorphisms in the HLA region related to depression, psoriasis, and schizophrenia. Nevertheless, given the polygenic nature of psoriasis, such associations may only occur in some patients. (ii) Considering the circumstances inherent to the disease itself (e.g., pain and itching, facial or genital wounds, stigmatization, sleep disturbance, deterioration on self-esteem), it can be recognized within adjustment disorders, a category that includes depressive symptoms caused by a stressor. (iii) The inflammatory state of the patient’s skin, as in psoriasis, might operate to exacerbate psychiatric disorders through synergistic routes present in both processes. Several studies on the relationship between psoriasis and mental disorders are presented in Table 3.

### 4.4. Chronic Wounds

Depression is a psychological condition commonly comorbid in people that suffer from chronic wounds [155]. Patients with chronic wounds frequently express a sense of lost autonomy in their daily lives due to the external management of their skin condition, and many patients are also pessimistic about their future prospects and about the potential for wound healing [156]. The pathophysiology of depression affects wound healing by modifying immune function, heightening inflammatory responses, and raising stress hormone levels, all of which collectively disrupt the natural capacity of the body for effective tissue repair [80]. The neuroendocrine system plays a central role in modulating how psychological stress affects wound healing. The secretion of stress hormones, including cortisol and catecholamines, as a reaction to psychological stress, can substantially hinder the healing process. Evidence indicates that cortisol can slow down wound repair by suppressing the inflammatory response, which is pivotal for the early stages of wound healing. Similarly, catecholamines impair healing by reducing blood circulation to the wound area [80,157]. Table 3 reviews various studies that link psychological disorders to chronic wounds.

## 5. Discussion and Remarks

The SM is formed by the association of several microbial taxa, including bacteria, fungi, and viruses. These microbial species shift during life and are located along body sites depending on the skin’s physicochemical and biochemical properties, forming distinct microenvironments or niches and establishing specific ecological relationships through trophic, competitive, and quorum-sensing mechanisms [6]. Compared to other microbiomes, such as the gut, the biomass, abundance, and biodiversity of the SM is relatively low [158,159] and varies significantly with individuality and ethnicity, which may be explained by endogenous (immune status, sweat, and sebum composition) and/ or exogenous (dietary and hygiene preferences) factors [5]. While distinct variations have been observed among different ethnic groups, it is still uncertain whether race and genetics determine the composition of the SM or whether it is a consequence of diet and lifestyle [160,161]. In this sense, the dietary habits prevalent in several geographical regions (such as the Western, Mediterranean, or vegetarian diets) influence the gut microbiome [162], a fact that can be hypothesized to occur also in the case of the SM.

The eubiosis of the SM may be altered by several extrinsic and intrinsic factors (Figure 1), provoking an imbalance in the SM and the production of skin diseases. In this sense, it is interesting to establish the role of commensal microorganisms in the SM, since these same microorganisms can act as opportunistic pathogens and initiate an infectious process under certain conditions and among immunocompromised subjects [4]. For example, *S. epidermidis* has been implicated in AD and in Netherton syndrome [163]; *Corynebacterium* spp. has been reported in various skin diseases such as erythrasma, keratolysis, and trichobacteriosis [164]; and *Malassezia* spp. has been associated with head and neck dermatitis, seborrheic dermatitis, pityriasis versicolor, and folliculitis [165]. All these opportunistic microorganisms promote immune tolerance while also eliciting pro-inflammatory responses [166]. The human innate immune system can be stimulated against *S. epidermidis* via Toll-like receptors that enhance antibacterial reactions, trigger inflammation, and result in the stimulation of immune system effectors, including type-1 interferon (IFN-α and IFN-β), pro-inflammatory cytokines (IL-1, IL-6, and TNF-α), and nitric oxide [167]. In the case of *Corynebacterium* spp. infection, particularly *C. accolens*, there is an increase in the number and activation of a defined subset of γδ T cells and IL-23 [168]. Finally, *Malassezia* spp. infection presents an immunological paradox; in some circumstances, it acts as an adjuvant, activating the complement cascade and eliciting both cellular and humoral immune responses in healthy individuals. In contrast, it also seems to have the ability not only to evade the immune system but also to suppress the response directed against it [169]. 

Therefore, questions arise such as: is it appropriate to call these microorganisms commensals, is there such a thing as a healthy SM, or what are the factors that trigger the commensal–pathogen shift? To provide answers, it is pivotal that forthcoming research focus on the investigation of understudied species and their molecular and biochemical mechanisms of interaction within their ecosystems [4]. Several skin diseases have already been associated with dysbiosis in the SM, where is a predominance of some microorganisms to the detriment of others is produced through the secretion of mediators that inhibit pathogen signaling systems, preventing their colonization and damage to host tissues. According to Townsend et al. [3], future work on the SM will likely continue to explore: (i) the age evolution of the SM and the factors involved; (ii) the complex interactions between microorganisms and the immune system and how they change over the lifespan; (iii) the pathogenesis of SM dysbiosis; and (iv) the development of new therapeutic strategies to effectively modulate the microbial balance.

In this review, we have shown that certain skin diseases (i.e., acne, AD, psoriasis, and chronic wounds) have a substantial impact on the patient’s quality of life and psychological wellness (Table 1, Table 2 and Table 3). Most of the studies point out that these skin diseases are closely linked to distressing mental states, including anxiety, depression, and stress, while in severe cases, suicidal ideation has also been reported as a consequence [105,119,120]. These results could be related to the fact that skin conditions, particularly those that are visible and chronic, can constitute a stigma, and that have profound social consequences for the affected individuals. Indeed, during adolescence, a critical life period for social and emotional development, these conditions may increase the risk of victimization through harassment dynamics (e.g., mocking, social exclusion). The visible nature of skin disorders such as acne, AD, or psoriasis can lead to negative peer reactions expressed through bullying, which can elicit adverse self-conscious emotional responses such as humiliation or shame, perturbing the personal and social well-being of individuals [170]. Thus, this kind of stigmatization is not merely superficial, especially because it intersects with the psychological vulnerability of these developmental stages, potentially contributing to lasting emotional trauma. In addition, the recalcitrant symptoms of these skin conditions may exacerbate the distress experienced, leading to a process where stress worsens the skin pathology, which in turn heightens the emotional and social burden. In this respect, overwhelming stress and isolation might drive affected youth toward maladaptive coping mechanisms, including the initiation of risky behaviors, in order to escape from reality, such as continued substance use [171]. Consequently, the psychosocial impact of skin diseases should be considered a pivotal component of patient care, emphasizing the need for holistic treatment approaches. Understanding the full scope of these interactions highlights the importance of integrating dermatological care with psychological support, particularly in pediatric and adolescent populations.

Psychodermatology is recognized to be a collaborative approach between dermatologists, psychiatrists, and psychologists to help those individuals with mental disorders that arise from a skin disease, and also those with a skin disease that is influenced by mental disorders. This multidisciplinary framework is thought to yield better outcomes compared to medical treatment individually [172]. Psychodermatologic conditions can be divided into three groups: psychophysiological disorders, psychiatric disorders with dermatologic symptoms, and dermatologic disorders with psychiatric symptoms. The term “psychophysiological disorder” describes skin conditions that are exacerbated by emotional stress (e.g., acne, eczema, and psoriasis). In cases of primary psychiatric disorders, the primary issue is psychological, with skin manifestations being secondary and sometimes self-induced. In cases of secondary psychiatric disorders, the skin lesions themselves lead to negative effects on the patient’s body image, mood, and self-esteem. This can result in feelings of frustration, shame, and social anxiety [173]. The effect of the skin disease on the patient’s quality of life is considered a stronger predictor of psychiatric morbidity than the clinical severity of the skin condition. On the other hand, it is crucial to consider the side effects of skin medication on the psychological well-being of patients. For instance, Ding et al. [174] studied isotretinoin, a vitamin A derivative employed for the treatment of acne, in relation to the potential psychological consequences experienced by patients due to its use. These authors concluded that these side effects remain unclear and do not seem to differ significantly from the psychological effects caused by acne itself.

Reported risk factors for skin disease include socioeconomic aspects such as bacterial infections, dietary variables, excess of body weight, frequent skin touching, humid environments, stress, tobacco use, topical oily products that obstruct pores, as well as genetic and hormonal factors [175]. The “hygiene hypothesis” postulates that insufficient exposure to a diverse array of skin microbiota during childhood may impair the proper “training” of the immune system [176]. This lack of exposure is thought to diminish the immune system’s resistance to microbial pathogens, thereby increasing susceptibility to infections and to other diseases. AD and other atopic skin diseases may be associated with an overly sterile (abiotic) environment [177].

There is a bidirectional relationship between gut and skin dysbiosis, with imbalances in the gut microbiome influencing the pathophysiology of various inflammatory skin conditions [178]. Therefore, in the future, the treatment of skin diseases and their psychological consequences can be based on the adjustment of human skin and gut microbiomes with healthy dietary patterns and with probiotics, prebiotics, synbiotics, and fecal microbiota transplantation [179,180,181], as well as with other strategies that are providing promising results (e.g., skin microbiota transplantation) [182]. In this regard, engineering and restoration of skin microbiota could emerge as a potent strategy for treating skin diseases in the future [183]. Finally, it is important to highlight that the prevalence of skin diseases has increased in recent years, with fungal infections being major contributors to skin and subcutaneous diseases worldwide [184]. Additionally, several environmental factors and food additives appear to be directly or indirectly involved in the rising incidence of these health conditions [185,186].

## Figures and Tables

**Table 1:** Relationship between acne vulgaris and psychological disorders.

Study/Country	Intervention	Instruments	Main Outcomes
Eyüboglu et al. [101]/India	*N* = 164 patients with acne (age: 12–17). *N* = 188 controls.	CDLQI, PedsQL, SDQ, GAGS.	Acne had an impact on the psychosocial life of patients. A worsening of quality of life was not affected by duration, severity of acne, and age.
Kodra et al. [102]/Albania	*N* = 65 patients with acne (age: 13–19). *N* = 60 controls. Cross-sectional study.	GAGS, RSES, HADS.	A significant correlation was obtained between poor self-esteem, anxiety, and the severity of acne. No correlation was found between depression and the severity of acne.
How and Shamsudin [103]/Malasya	*N* = 55 patients with acne (mean age: 23.2); 70.9% females. Cross-sectional study.	CASS, CADI, DASS-21.	Most of acne patients reported feeling anxious, depressed, and stressed.
Mostafa et al. [104]/Egypt	*N* = 200 patients with acne (age: 12–15). Descriptive correlational design.	BDI, RSES, BIS.	There was a significant relationship between acne and depressive symptoms, body image, and self-esteem.
Altunay et al. [105]/13 European countries	*N* = 213 patients with acne (mean age: 24.6); 62% females. *N* = 213 controls. Multicentered observational cross-sectional study.	HADS, DLQI, EuroQoL5D3L.	Patients with acne showed higher scores for anxiety and depression compared to controls. More than 10% of patients had suicidal ideation, and 4% of them implied that acne was the cause of their suicidal thoughts.
D Kandre et al. [106]/India	*N* = 46 patients with acne (mean age: 20.0); 27% females. Cross-sectional study.	GAGS, DASS-21.	Patients with acne presented higher and significantly higher scores of stress, anxiety, and depression. Anxiety and depression were correlated significantly with acne duration.
Rafiq and Mehdi [107]/Pakistan	*N* = 100 patients with acne (mean age: 21.0); 58% females. Cross-sectional study.	DASS-21, GAGS.	A significant impact of acne vulgaris in the frequency of depression, anxiety, and stress.
Molla et al. [108]/Saudi Arabia	*N* = 296 patients with acne (age: 12–60); 67.6% females. *N* = 296 controls. Case–control study for 3 months.	HADS, GAGS.	A significant difference with the control group was found for overall anxiety. No significant difference was obtained in the depression score. A positive correlation between anxiety and depression scores in patients with acne.
Khan et al. [109]/Saudi Arabia	*N* = 476 patients with acne (age: 15–25). Cross-sectional study.	DLQI.	Acne affected the quality of life of young females.
Rayapureddy et al. [110]/India	*N* = 100 patients with acne (mean age: 24.5); 68% females. Cross-sectional study.	GAGS, HADS.	A significant association was found between the severity of acne and anxiety and depression.
Agarwal et al. [111]/India	*N* = 660 patients with acne (mean age: 18.1); 28.5% females. Cross-sectional study.	GHQ, BDI, HAMA.	About 50% of acne patients presented psychological morbidity, including different severities of depression and anxiety. Acne significantly affected self-esteem and confidence of the youth.
Kunjumon et al. [112]/India	*N* = 254 patients with acne (mean age: 24.9); 70% females. Cross-sectional study.	HAM-D, LSAS.	There was a significant higher incidence of social anxiety and depression among the patients with acne vulgaris.
Morshed et al. [113]/Bangladesh	*N* = 150 patients with acne (mean age: 21.7); 60% females. Cross-sectional study.	CADI, RSES, GAGS, DLQI, DASS-21, WHOQoL.	Positive and significant correlations between acne and depression and anxiety and stress. A negative impact of acne on patient’s self-esteem and quality of life was found.
Noor et al. [114]/Pakistan	*N* = 102 patients with acne (mean age: 21.5); 88.2% females. Cross-sectional study.	GAGS, DASS-21.	A strong and significant correlation was observed between acne and stress, anxiety, and depression.
Sharma et al. [115]/India	*N* = 400 patients with acne (age: >13). Cross-sectional study.	CADI, HADS, WHOQoL, GAGS.	Acne significantly impacted the quality of life and mental health of patients.
Tasneem et al. [99]/Bangladesh	*N* = 185 patients with acne (mean age: 22.5); 83.8% females. Cross-sectional study.	CADI, IGA, BDI.	Patients with moderate and severe acne had a higher likelihood of depressive symptoms. More than 65% of participants reported that they had depressive symptoms.
Karaagac et al. [100]/Turkey	*N* = 81 patients with acne (mean age: 22.9); 74.1% females. Cross-sectional study.	DASS-21, TFEQ-21.	No relationship was found between acne severity and depression or anxiety. These conditions can increase the risk of eating disorders among acne patients.
Leskelä et al. [116]/Finland	*N* = 150 patients with acne (mean age: 46); 53.7% females. *N* = 1907 controls. Longitudinal study.	BDI, AIS, GAD-7, STAI; 15D-HRQoL.	Cases with acne presented more depressive symptoms. The severity of acne did not correlate with psychological symptoms.
Shakir et al. [117]/Pakistan	*N* = 327 patients with acne (age: 12–19); 44.6% females. Cross-sectional study.	CADI, RSES, T-QoL, SAAS.	Significant correlations were found between acne and social appearance, anxiety, self-esteem, and quality of life. Acne significantly impacts adolescents’ psychological well-being, increasing social appearance anxiety and reducing self-esteem.

AIS: Athens Insomnia Scale; BDI: Beck Depression Inventory; BIS: Body Image Scale; CADI: Cardiff Acne Disability Index; CASS: Comprehensive Acne Severity Scale; CDLQI: Children’s Dermatology Life Quality Index; DASS-21: Depression–Anxiety–Stress Scale; DLQI: Dermatology Life Quality Index; EuroQoL5D3L: EQ-5D-3L, General Health Status—EQ-VAS, Visual Analog Scale; GAD-7: Generalized Anxiety Disorder; GAGS: Global Acne Grading System; GHQ: Goldberg’s Health Questionnaire-12; HADS: Hospital Anxiety and Depression Scale; HAMA: Hamilton Rating Scale for Anxiety; HAM-D: Hamilton Depression Rating Scale; 15D-HRQoL: 15 Dimensional Measure of Health-related Quality of Life; IGA: Investigator’s Global Assessment Scale; LSAS: Liebowitz Social Anxiety Scale; PedsQL: Pediatric Quality of Life Questionnaire; RSES: Rosenberg Self-Esteem Scale; SAAS: Social Appearance Anxiety Scale; SDQ: Strength and Difficulties Questionnaire; STAI: State-Trait Anxiety Inventory; TFEQ-21: Three-Factor Nutrition Questionnaire; T-QoL: Teenager’s Quality of Life Index; WHOQoL: WHO Quality of Life Scale.

**Table 2:** Relationship between atopic dermatitis and psychological disorders.

Study/Country	Intervention	Instruments	Main Outcomes
Kim et al. [122]/Korea	*N* = 1517 patients with AD (mean age: 19.8). *N* = 118,991 healthy participants. Cross-sectional study.	KMPI.	Moderate to severe AD was significantly related to depression, anxiety, and somatization.
Catal et al. [123]/Turkey	*N* = 80 patients with AD (mean age: 48.4); 52.5% females. *N* = 74 controls. Multicentered study.	ECI-4.	ADHD, conduct disorder, anxiety, eating disorder and tic disorder symptom severity were found to be higher in patients with AD compared to control group.
Eckert [124]/Five European countries	*N* = 1860 patients with AD (mean age: 43.9); 70.5% females. *N* = 80,600 controls. Prospective study.	DLQI, MCS, HRQoL, PCS.	Depression and anxiety were common in patients with AD versus controls.
Ring et al. [125]/Germany	*N* = 1189 patients with AD (age: 18–87); 56% females. Observational study.	POEM, DLQI, AESEC, HADS.	Adults with a moderate to severe forms of AD presented higher psychological suffering.
Schonmann et al. [126]/United Kingdom	*N* = 526,808 patients with AD (mean age: 44). *N* = 3,095,838 controls. Longitudinal cohort study (1998–2016).		Treated AD was associated with an increase in the risk of newly diagnosed depression and anxiety. Strong evidence for a dose–response association was observed between depression and AD. Individuals affected with AD were more likely to develop depression and anxiety, regardless of AD severity.
Talamonti et al. [127]/Italy	*N* = 174 patients with AD (mean age: 38.1); 51.1% females. *N* = 178 controls. Cross-sectional study.	DLQI, BDI, TAS-20, EASI, PNRS.	A high prevalence of alexithymic personality features in AD patients compared to controls. AD has a significant impact on psychosocial well-being and quality of life.
Keller et al. [128]/Germany	*N* = 1764 patients with AD (age: 3–10); *N* = 937 patients with AD (11–18); *N* = 915 patients with self-reported AD; 46.9% females. Cross-sectional study.	SDQ, SES.	In younger children, AD was associated with internalizing and externalizing problems. Parents of adolescents were more likely to perceive associations between AD and behavioral difficulties than the adolescents themselves.
Kern et al. [129]/United Kingdom	*N* = 1356 patients with AD; 48.8% females. *N* = 11,181 controls. Longitudinal cohort study (1990–2009).	SDQ, SMFQ, DAWBA.	Severe AD was associated with symptoms of depression and internalizing behaviors throughout childhood and adolescence.
Muzzolon et al. [130]/Brazil	*N* = 100 patients with AD (mean age: 6.5); 55% females. *N* = 50 controls (mean age: 8.4). Cross-sectional study.	CBCL.	Children and adolescents with AD presented a high risk of mental disorders. Their healthy siblings also presented impairment in their mental health.
Iannone et al. [131]/Italy	*N* = 16 patients with AD (mean age: 46); 51.2% females. *N* = 64 controls. Cross-sectional study.	DLQI, HRQoL.	Generalized anxiety and depressive disorders with anxious distress were found to be risk factors for AD.
Vinh et al. [132]/Vietnam	*N* = 208 patients with AD (age: >18); 59.1% females. 46.2% patients with autoimmune–allergic comorbidity. Cross-sectional study.	HADS, DCAD.	Patients with severe AD were more prone to anxiety. Other disorders, such as allergies, autoimmune diseases, pruritus, and insomnia also presented correlation with anxiety and depression disorders.
Ferrucci et al. [133]/Italy	*N* = 66 patients with AD (42 treated for 1 year with a human mAb [mean age: 37.7]); 45.5% females.	POEM, PGA, EASI, DLQI, HADS, SQ-NRS, IAT, DAST, itch-INRS.	Patients with AD presented significant symptoms of anxiety, depression, and internet addiction. Severity of anxiety and depression was directly proportional to severity of AD. Patients with AD treated for 1 year with monoclonal antibody showed a better mental health profile (less severe anxiety and depression).
Johnson et al. [134]/United States	*N* = 954 patients with AD (adults *N* = 795 [mean age: 38]; 68.2% females) (children and adolescents *N* = 159 [mean age: 11.4]; 42.1% females). Online survey.	HADS.	The severity of AD impacted the perception of the relationship between AD and mental health.

AESEC: Atopic Eczema Score of Emotional Consequences; BDI: Beck Depression Inventory; CBCL: Child Behavior Checklist; DAST: Drug Abuse Screening; DAWBA: Development and Well-Being Assessment; DCAD: Diagnostic Criteria for Atopic Dermatitis Criteria; DLQI: Dermatology Life Quality Index; EASI: Eczema Area and Severity Index; ECI-4: Early Childhood Inventory-4; HADS: Hospital Anxiety and Depression Scale; HRQoL: Health-Related Quality of Life; IAT: Internet Addiction Test; itch-INRS: Itch Numerical Rating Scale; KMPI: Korean Military Multiphasic Personality Inventory; MCS: Mental Component Summary; PCI: Physical Component Summary; PGA: Physical Global Assessment; PNRS: Pruritus Numerical Rating Scale; POEM: Patient-Oriented Eczema Measure; SDQ: Strength and Difficulties Questionnaire; SES: Socioeconomic Status; SMFQ: Short Moods and Feelings Questionnaire; SQ-NRS: Sleep Quality Numeric Rating Scales; TAS-20: Toronto Alexithymia Scale.

**Table 3:** Association of psychological disorders with psoriasis (P) and chronic wounds.

Study/Country	Intervention	Instruments	Main Outcomes
Jensen et al. [138]/Denmark	*N* = 179 patients with P (age: 18–82); 35.8% females. *N* = 105 controls. Cross-sectional study.	PASI, DLQI, ISI, PSQI, PSS, BDI, Itch.	About 25% of the psoriasis patients reported clinical insomnia. Itch was the main predictor of impaired sleep.
Egeberg et al. [139]/Denmark	*N* = 45,641 patients with P (mean age: 51.1); 53.3% females. Longitudinal cohort study.	PASI, BDI.	Patients with psoriasis, especially those who received therapy and were aged 40–50 years have a small significant increased risk of depression. Psoriasis was independently associated with risk of depression.
Aguayo-Carreras [140]/Spain	*N* = 130 patients with P (mean age: 46.8); 42% females. Cross-sectional study.	DS14, SASS, HADS, MGH-SFQ, SFHS, PASI, PDI.	Patients with psoriasis and type D personality presented a high risk of depression and anxiety. Patients had a worse quality of life, more problems of sleep, poorer social adaptation, and higher frequency of sexual disturbances.
Mohapatra et al. [141]/India	*N* = 149 patients with P (mean age: 38.9); 45.6% females. Cross-sectional study.	PASI, BDI, HAM-A, SRQ-20.	Almost all the psoriasis patients had a mild degree of anxiety symptoms, but only 40% presented mild to severe depression. The majority of the patients established a decreasing order of frequency in the following domains: embarrassment, discomfort, fear, anger, physical limitation, depression, and cognitive impairment.
Bakar et al. [142]/Malaysia	*N* = 174 patients with P (mean age: 46.4); 46% females. Cross-sectional study.	DLQI, HADS, PASI.	People with psoriasis exhibited notable symptoms of depression, anxiety, and poor quality of life.
Oh et al. [143]/Korea	*N* = 10,868 patients with P (age: 16–74). *N* = 620,055 controls. Cohort study, follow-up 12 years.	DLQI, BDI, PASI, PHQ-15.	An increased risk of depression and somatoform disorders were found in the experimental group compared to the referent cohort. The degree and severity of psoriasis were positively correlated with a higher risk of developing depression and somatoform disorders.
Pollo et al. [144]/Brazil	*N* = 281 patients with P (mean age: 52.1); 52% females. Cross-sectional study.	HADS, DLQI, PASI.	The prevalence of anxiety and depression symptoms was low. Female gender, income, age, illness length, and quality of life were associated with anxiety and depression scores in patients with psoriasis.
Badaik et al. [145]/ Canada	*N* = 15,100 patients with P (mean age: 52.6); 56% females. *N* = 75,500 controls. Longitudinal cohort study.	ECT, BDI.	Psoriasis patients presented higher scores for anxiety and depression compared to controls. The greatest risk of anxiety and depression among patients was in the 0 to 20 age group, mainly in females.
da Silva et al. [146]/Germany	*N* = 138 patients with P (mean age: 46.3); 46.7% females. From total: *N* = 64 without pruritus, *N* = 43 with moderate/severe pruritus; *N* = 31 with anogenital psoriasis. *N* = 76 controls. Cross-sectional study.	PASI, DLQI, GAD-2, DCQ, PHQ, PBI, Itchy, PSQ, RSS.	Patients with moderate/severe pruritus reported more quality of life impairments, depression, anxiety, and dysmorphic concerns. Moderate/severe pruritus had a deleterious effect on depression and stigmatization for patients with anogenital psoriasis.
Iannone et al. [131]/Italy	*N* = 20 patients with P (mean age: 46). Cross-sectional study.	EASI, PASI, DLQI.	Psoriasis was associated with psychiatric disorders. Major depressive disorder showed a specific association with DLQI.
Schuster et al. [147]/Germany	*N* = 722 patients with P (mean age: 45.8); 62.7% females. Cross-sectional study.	SPANE, SWLS, WHO-WBI-5.	Participants with psoriasis reported lower subjective well-being compared to the general population. The severity of the disease was associated with low subjective well-being and depression.
Adesanya et al. [148]/United Kingdom	*N* = 36,321 patients with P (age: >18 and <60). *N* = 1,801,875 controls. Longitudinal cohort study, follow-up 5.9 years (mean).	CMI.	Psoriasis was associated with an increased hazard of several mental illnesses (schizophrenia, BD, other psychoses). After adjusting by mediating factors, the mental illness risk attenuated.
Finlayson et al. [149]/United States-Australia	*N* = 247 patients with chronic wounds (mean age: 69.1); 49% females. Retrospective study.	MOSPM, CWISDS GDS, HRQoL, SFHS.	Significant relationships were found between delayed ulcer healing, decreased quality of life, and inclusion in the severe symptom subgroup. The severe symptoms subgroup reported moderate-to-severe levels of pain, depression, fatigue, or sleep disturbances.
Walburn et al. [150]/United Kingdom	*N* = 63 patients with chronic wounds (mean age: 68.1); 60.3% females. Retrospective observational study.	PSS, HADS, RIPQ, SDSCA, SFHS, HRQoL, IPQ-R.	Psychological and sociodemographic factors were associated with healing.
Fauziyah and Gayatri [151]/Indonesia	*N* = 76 patients with chronic wounds. Cross-sectional study.	NRS, QSC-R23, PSQI.	Moderate–severe pain and poor sleep quality produced a higher stress level. The relationship between pain and sleep quality was not directly correlated by the influence of stress.
Fino et al. [152]/Italy	*N* = 33 patients with chronic wounds (mean age: 71). *N* = 33 controls. Cross-sectional study.	BDI.	The depression among patients with chronic wounds had a multifactorial origin. The improvement of psychoemotional state meant better patient compliance.
Dantas et al. [153]/Brazil	*N* = 85 patients with chronic wounds (age: >60 [45.9%]); 52.9% females. Cross-sectional study.	HR-QoL, SFHS, CWIS.	Chronic wounds for many years can cause physical (pain, insomnia, and lack of appetite), psychological (low self-esteem, apathy, lack of motivation), and social (isolation, inability to work) harms, which have repercussions on quality of life and negatively affect to the possibility of a cure.
Spoer et al. [154]/United States	*N* = 265 patients with chronic wounds (mean age: 66); 38.9% females. Cross-sectional study.	LEFS, SFHS, SRQ-20, PROMIS-3a.	Patients with lower extremity wounds experienced potentially meaningful psychological distress (depression and anxiety). There were no differences in physical or social quality of life in patients.

BDI: Beck Depression Inventory; CMI: Charlson Comorbidity Index; CWIS: Cardiff Wound Impact Schedule; CWISDS: Cardiff Wound Impact Schedule Disturbed Sleep; DCQ: Dysmorphic Concern Questionnaire; DLQI: Dermatology Life Quality Index; DS14: Type D Scale-14; EASI: Eczema Area and Severity Index; ECT: Electroconvulsive Therapy; GAD-2: Generalized Anxiety Disorder-2; GDS: Geriatric Depression Scale; HADS: Hospital Anxiety and Depression Scale; HAM-A: Hamilton Anxiety Rating Scale; HRQoL: Health-Related Quality of Life; IPQ-R: Illness Perception Questionnaire—Revised; ISI: Insomnia Severity Index; Itch: Itch Severity Scale; LEFS: Lower Extremity Functional Scale; MGH-SFQ: Massachusetts General Hospital—Sexual Functioning Questionnaire; MOSPM: Medical Outcome Study Pain Measure; NRS: Numeric Rating Scale; PASI: Psoriasis Area Security Index; PBI: Patient Benefit Index; PDI: Psoriasis Disability Index; PHQ: Patient Health Questionnaire; PHQ-15: Somatic Symptom Severity Scale; PROMIS-3a: Patient-Reported Outcomes Measurement Information System Scale; PSQ: Perceived Stigmatization Questionnaire; PSQI: Pittsburg Sleep Quality Index; PSS: Perceived Stress Scale; QSC-R23: Questionnaire on Stress in Cancer Patients-Revised; RIPQ: Revised Illness Perception Questionnaire; RSS: Relationship and Sexuality Scale; SASS: Self-Applied Scale of Social Adaptation; SDSCA: Summary of Diabetes Self-Care Activities; SFHS: Short Form Health Survey; SPANE: Scale of Positive and Negative Experience; SRQ-20: Self Reporting Questionnaire-20; SWLS: Satisfaction With Life Scale; WHO-WBI-5: WHO Well Being Index-5.

## Data Availability

Not applicable.
